# A Novel Metabolic Reprogramming Strategy for the Treatment of Diabetes‐Associated Breast Cancer

**DOI:** 10.1002/advs.202102303

**Published:** 2022-01-12

**Authors:** Qiongyu Hao, Zhimin Huang, Qun Li, Dingxie Liu, Piwen Wang, Kun Wang, Jieqing Li, Wei Cao, Wenhong Deng, Ke Wu, Rui Su, Zhongmin Liu, Jay Vadgama, Yong Wu

**Affiliations:** ^1^ Division of Cancer Research and Training Department of Internal Medicine Charles Drew University of Medicine and Science David Geffen UCLA School of Medicine and UCLA Jonsson Comprehensive Cancer Center Los Angeles CA 90095 USA; ^2^ Key Laboratory of Cell Differentiation and Apoptosis Ministry of Education Department of Pathophysiology Shanghai Jiao‐Tong University School of Medicine Shanghai 200025 China; ^3^ Department of Oncology Shanghai East Hospital School of Medicine Tongji University Shanghai 200123 China; ^4^ Bluewater Biotech LLC New Providence NJ 07974 USA; ^5^ Department of Breast Cancer Cancer Center Guangdong Provincial People's Hospital & Guangdong Academy of Medical Sciences Guangzhou 510080 China; ^6^ Department of General Surgery Renmin Hospital of Wuhan University Wuhan 430060 China; ^7^ College of Engineering University of California Berkeley CA 94720 USA; ^8^ The Institute for Biomedical Engineering & Nano Science Shanghai East Hospital Tongji University School of Medicine Shanghai 200120 China; ^9^ Department of Bioengineering Rice University Houston TX 77005 USA

**Keywords:** breast cancer, diabetes, lactate, MCT4, metabolic reprogramming, metformin

## Abstract

Diabetes is directly related to the risk of breast cancer (BC) occurrence and worsened BC prognosis. Currently, there are no specific treatments for diabetes‐associated BC. This paper aims to understand the fundamental mechanisms of diabetes‐induced BC progression and to develop personalized treatments. It reports a metabolic reprogramming strategy (MRS) that pharmaceutical induction of glucose import and glycolysis with metformin and NF‐*κ*B inhibitor (NF‐*κ*Bi) while blocking the export of excessive lactate via inhibiting monocarboxylate transporter 4 (MCT4) leads to a metabolic crisis within the cancer cells. It demonstrates that the MRS shifts the metabolism of BC cells toward higher production of lactate, blocks lactate secretion, prompts intracellular acidification and induces significant cytotoxicity. Moreover, a novel MCT4 inhibitor CB‐2 has been identified by structure‐based virtual screening. A triple combination of metformin, CB‐2, and trabectedin, a drug that impedes NF‐*κ*B signaling, strongly inhibits BC cells. Compared to normal glucose condition, MRS elicits more potent cancer cell‐killing effects under high glucose condition. Animal model studies show that diabetic conditions promote the proliferation and progression of BC xenografts in nude mice and that MRS treatment significantly inhibits HG‐induced BC progression. Therefore, inhibition of MCT4 combined with metformin/NF‐*κ*Bi is a promising cancer therapy, especially for diabetes‐associated BC.

## Graphical abstract

A metabolic reprogramming strategy (MRS) that pharmaceutical induction of glucose import and glycolysis to even higher levels while blocking the export of excessive lactate leads to a metabolic crisis within the cancer cells. MRS can be realized by pharmacological inhibition of MCT4 combined with metformin/NF‐*κ*Bi, which is a promising cancer therapy, especially for diabetes‐associated breast cancer.

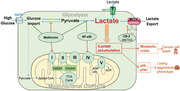

## 1 Introduction

Diabetes is a growing public health problem that is directly related to the risk of breast cancer (BC) occurrence ^[^ ^1^ ^]^ and worsened BC prognosis ^[^ ^2^ ^]^. For example, 5‐year mortality rates were significantly higher in patients diagnosed with both breast cancer and diabetes than in comparable breast cancer patients without diabetes ^[^ ^3^ ^]^. In addition, BC treatment can lead to weight gain resultant diabetes ^[^ ^4^ ^]^. There exist some important distinctions between the BC patients with and without diabetes in the regimen selection and outcomes of cancer therapy ^[^ ^5^ ^]^. Currently there are no specific treatments to target diabetes‐associated BC. Our goals are to understand the fundamental mechanisms of diabetes‐induced BC progression, and to develop personalized treatments for diabetes‐associated BC.

Main pharmaceutical approaches for cancer treatment are based on targeting the differences between cancer and normal cells. One of these essential differences is the distorted glucose metabolism in cancer cells, i.e., upregulation of aerobic glycolysis ^[^ ^6^ ^]^. Thus, this unique metabolic alteration in malignant cells may be exploited to develop promising therapeutic strategies, i.e, pharmacological inhibition of glycolysis. However, glycolysis inhibition rarely causes cell death ^[^ ^7^ ^]^ and the implementation of the glycolytic inhibitor, e.g., 2‐DG as an anticancer agent has been a disappointment ^[^ ^8^ ^]^.

Here, we are considering a paradigm shift in which, contrary to blocking glycolysis, we aggravate it, particularly in a hyperglycemic condition. The central hypothesis of this strategy is that pharmaceutical induction of glucose import and glycolysis to even higher levels while blocking the products of glycolysis from entering the tricarboxylic acid (TCA) cycle, results in production of high amounts of lactate. Meanwhile, blocking the export of excessive lactate leads to a metabolic crisis and intracellular acidification within the cancer cells, causing their death. Our preliminary results indicate that this metabolic reprogramming strategy (MRS) is feasible by blocking lactate export via inhibiting monocarboxylate transporter 4 (MCT4) in combination with promoting lactate production via forcing glycolysis with metformin and NF‐*κ*B inhibitor (NF‐*κ*Bi) treatment, leading to an accumulation of intracellular lactate (iLactate) and a decrease in intracellular pH (pHi). This type of combination therapy is promising because it can rapidly disable tumor cell growth, but with a minor effect on normal cells. Moreover, we have identified CB‐2 as a novel small molecule MCT4 inhibitor (MCT4i) (Patent Pub. No.: US20190352282A1). Considering the general adverse effect of hyperglycemia in treatment of diabetic cancer patients, our proposed approach to target cancer metabolism has the advantage of possibly being even more effective in cancer patients with diabetes and high blood glucose.

The identification of CB‐2 as a MCT4 inhibitor can serve as an initial point for further development in this therapeutic target class. Moreover, the reasonable combination of metformin with NF‐*κ*Bi and MCT4i holds promise as an anticancer treatment. Together, our studies have a broad impact on the field by providing a novel idea for therapies targeting cancer cell metabolism. In the long term, these studies may reveal an effective therapeutic strategy for diabetes‐associated BC.

## 2 Results

### 2.1 The Metabolic Reprogramming Strategy (MRS) Shifts the Metabolism of BC Cells toward Higher Production of Lactate, Blocks Lactate Secretion, and Induces Intracellular Acidification

**Figure 1:**
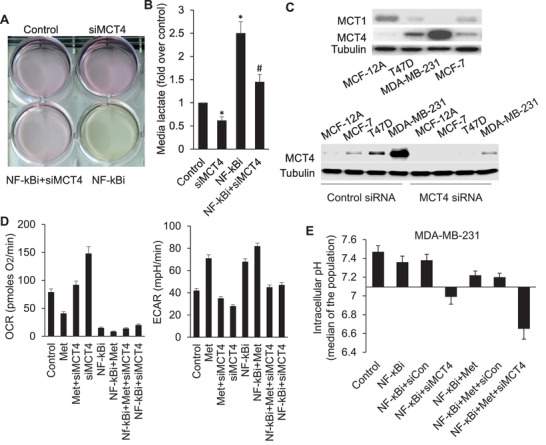
MCT4 expression is required for regulation of intracellular pH and extracellular acidification. A) MCF‐7 cells were transfected with Control or MCT4 siRNA for 24 h followed by incubation with NF‐*κ*Bi (IMD‐0354, 125 × 10^−6^ m) for 24 h. Acidification of the culture medium was evaluated by visually inspecting the color of the medium. B) Levels of lactate in the culture medium was then measured and normalized to cell number. *p < 0.05 versus control; #*p* < 0.05 versus NF‐*κ*Bi; C) MCT1 and MCT4 protein expressions (upper panel); Gene knockdown efficiency of MCT4 siRNA was verified from protein levels by Western analysis (lower panel). Data are representative of three independent experiments. D) The effect of MCT1 or MCT4 siRNA on OCR and ECAR in MDA‐MB‐231 cells was measured with a XF96 Extracellular Flux Analyzer (Seahorse/Agilent). Data are presented as the mean ± SD from six technical replicates. *p < 0.05 versus control. E) MDA‐MB‐231 cells were treated with NF‐*κ*Bi or together with metformin (Met, 0.75 × 10^−3^ m) or Control/MCT4 siRNA for 24 h. Intracellular pH was determined with a pH‐sensitive fluorescent probe SNARF‐4F.

Apart from coordinating many of the signals that drive proliferation during immunity, inflammation and oncogenesis, NF‐*κ*B regulates the metabolic reprogramming required for cell division during these processes. NF‐*κ*B inhibition causes cellular reprogramming to aerobic glycolysis ^[^ ^9^ ^]^. Here, treatment with the NF‐*κ*Bi induced an increase in secretion of lactate from breast cancer cell line, MCF‐7, and acidification of culture media. However, the secretion of lactate from the cells could be blocked by the gene knockdown of lactate transporter MCT4 with its specific siRNA (**Figure** **1**A,B). Interestingly, different breast cancer cell lines expressed different levels of MCT4 and MCT1. The MDA‐MB‐231 cell line expressed significantly higher levels of MCT4 (Figure 1C). The effect of MRS could be detected by checking OCR (oxygen consumption rate) and ECAR (extracellular acidification rate). Both NF‐*κ*Bi and metformin reduced the OCR significantly, induced ECAR and exhibited indications of a switch from oxidative phosphorylation to glycolysis. However, MRS treatment (NF‐*κ*Bi+metformin+MCT4 inhibition) resulted in a lower OCR and the block of ECAR, which can be interpreted as accumulation of lactate in the cells (Figure 1D). Accordingly, MRS treatment led to significant intracellular acidification with a strikingly decreased pHi (Figure 1E), which was further confirmed in the experiments using fluorescent BCFL‐AM as the probe for detecting pHi (Figure S1, Supporting Information).

### 2.2 The MRS Induces Significant Cytotoxicity in Breast Cancer Cells

**Figure 2:**
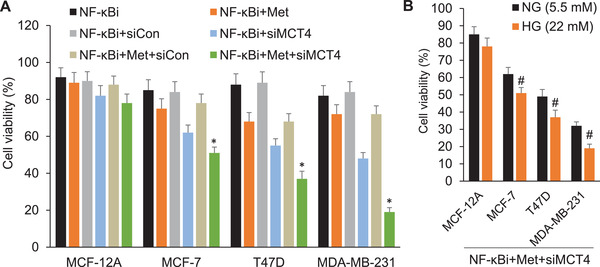
The significant growth inhibitory effect of MRS in MCF‐7, T47D, and MDA‐MB‐231 cells. A) MCF‐12A and breast cancer T47D, MDA‐MB‐231, and MCF‐7 cells under a HG condition were treated with NF‐*κ*Bi (IMD‐0354, 125 × 10^−6^ m) or together with metformin (Met, 0.75 × 10^−3^ m) or Control siRNA/MCT4 siRNA for 4 days. Forty‐eight hours post‐transfection, cells were subjected with a second round of siRNA transfection. After 4 days of incubation, the growth inhibition was detected by MTT assay. *p < 0.05 versus MCF‐12A. B) Comparison of growth inhibitory effects of MRS (NF‐*κ*Bi+Met+siMCT4) between normal glucose (NG, 5.5 × 10^−3^ m) and HG (22 × 10^−3^ m) conditions in different cell lines. #*p* < 0.05 versus NG.

To determine the cytotoxic effect of MRS, cell viability was measured after culturing the cells in the presence of different combinations of NF‐*κ*Bi, metformin and/or MCT4 siRNA under a high glucose (HG) condition. **Figure** **2**A indicated an inability of MRS to decrease the viability of MCF‐12A cells. However, MRS decreased the cell viability in MCF‐7, T47D, and MDA‐MB‐231 cells, with MDA‐MB‐231 cells showing the highest sensitivity. This phenomenon may be explained by the highest level of MCT4 expression in MDA‐MB‐231 cells. Furthermore, compared with MCF‐12A, MRS also significantly inhibited the migration and invasion ability of MDA‐MB‐231 cells (Figure S2 and S3, Supporting Information).

Cancer cells have an increased glucose metabolism, which is primarily characterized by augmented glucose uptake and aerobic glycolysis, the process of conversion of glucose into pyruvate eventually resulting in the production of lactate. Thus, one can reason that under a HG condition cancer cells have a higher glycolytic rate and lactate production, and MRS would further promote this process and cause a sharp rise of intracellular lactate, leading to a therapy where cancer cells poison themselves. To preliminarily substantiate this hypothesis, we compared the efficacy of MRS under normal and HG conditions. As shown in Figure 2B, MRS had little effects on the normal MCF‐12A cells but exhibited remarkable cytotoxicity on MCF‐7, T47D, and MDA‐MB‐231 cells under both conditions. Importantly, compared to normal glucose condition, MRS elicited more potent cancer cell‐killing effects under HG condition. Moreover, MRS also showed stronger inhibitory effects on cell migration and invasion in MCF‐7 and T‐47D cancer cells under HG condition versus normal glucose (Figure S4, Supporting Information). Together, by using MRS treatment, we exploited the abnormal metabolic characteristics in cancer cells to kill them without affecting normal cells.

### 2.3 Identification of Novel MCT4 Inhibitors

Lactic acid export from glycolytic cells is predominantly mediated by MCT4. Though MCT4 is absent from most normal tissues, MCT4 expression is highly upregulated, and correlates with poor survival, in many cancer indications, including triple‐negative breast cancer (TNBC), glioma, colorectal, head and neck, prostate, liver and kidney cancer. MCT4 has been in the spotlight in the recent years a promising new target for cancer pharmacotherapy ^[^ ^10^ ^]^. To further clarify the clinical correlation between MCT4 and breast cancer, Cox regression and Kaplan‐Meier survival analysis were performed for the associations of MCT4 expression with recurrence risk of BC. As shown in Figure S7 in the Supporting Information, the expression of MCT4 was positively correlated with the recurrence risk of overall breast cancer significantly. Our preliminary results demonstrate that MRS, i.e., promotion of glycolysis‐mediated lactate production combined with inhibition of MCT4‐mediated lactate export, may be an effective strategy to treat diabetes‐associated BC. Unfortunately, no potent and selective MCT4 inhibitors have been described. Moderate to weak MCT4 inhibitors are known (e.g., phloretin and *α*‐CN‐4‐OH‐cinnamate); however, these compounds promiscuously inhibit a number of other transporters, including MCT1. Thus, there is a need for potent and selective MCT4 inhibitors for use in the treatment of cancers.

**Figure 3:**
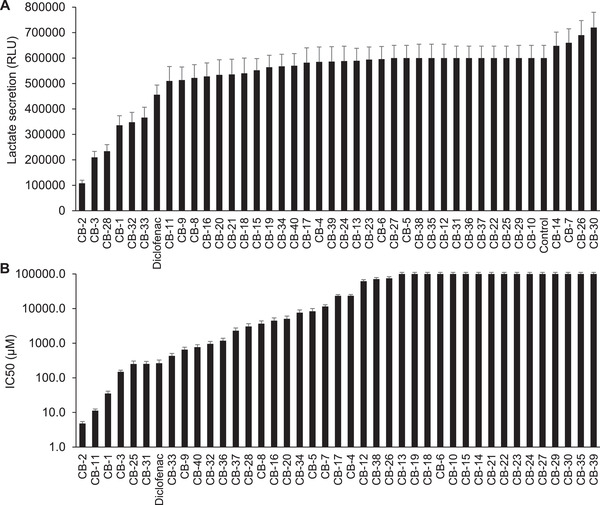
Cell‐based screening identifies novel MCT4 inhibitors suppressing lactate secretion and cell proliferation. A) Potential MCT4 inhibitors induced various levels of lactate secretion inhibition, with CB‐2, CB‐3 and CB‐28 being the most effective. MDA‐MB‐231 cells were incubated with various compounds (2.5 × 10^−6^ m) for 48 h followed by lactate secretion assay. B) MCT4 inhibitor candidates showed varying inhibitory effects on cell proliferation, with CB‐2, CB‐11, and CB‐1 showing the most profound inhibitions (IC50: 4.8 × 10^−6^, 11.3 × 10^−6^, and 35.3 × 10^−6^ m). Diclofenac was used as a positive control.

**Figure 4:**
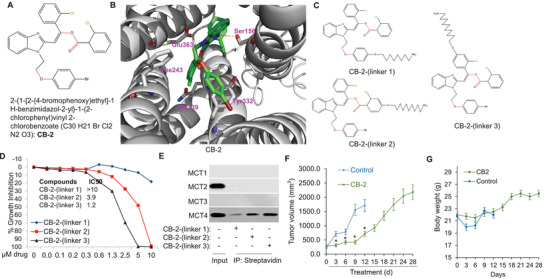
CB‐2 is a small‐molecule inhibitor of the MCT4. A) Chemical structure of CB‐2. B) Predicted binding mode of CB‐2 to MCT4. MCT4 is displayed as a gray cartoon, and CB‐2 is shown in green stick. The residues within the binding site of CB‐2 are highlighted as the stick representation. Hydrogen bond is indicated by dotted lines. C) The chemical structures of biotinylated CB‐2 with different position of linkers. D) IC50 curves for MDA‐MB‐231 cells treated with three different Biotin‐CB‐2s. E) MCT4 associates with Biotin‐CB‐2. Cell lysate was incubated with three different Biotin‐CB‐2s, followed by streptavidin pull‐down assay. The level of MCT1–4 associated with Biotin‐CB‐2s was analyzed through Western‐blotting analysis. F) MDA‐MB‐231 cells were injected subcutaneously into the flanks of female athymic nude mice. After the tumors reached a volume of 50 mm^3^, the mice were administered with CB‐2 (20 mg kg^−1^ day^−1^; i.p) or its vehicle for 4 weeks. The control groups were injected with the vehicle solution. Tumor volumes were measured with a caliper. G) Body weight of CB‐2‐treated mice or control mice was assessed during the experiment. Data are presented as mean ± SD (*n* = 8). *p < 0.05 versus Control; #*p* < 0.05 versus CB‐2.

Here, we therefore aimed to identify novel MCT4 inhibitors by structure‐based virtual screening, which has become an integral part of the drug discovery process. After generating the 3D model of human MCT4 structure using the I‐TASSER On‐line Server, we screened some of the best active compounds and verified their activities against MCT4. A total of 237 (113 from enamine; 84 from chemdiv, 40 from Chembridge) potential hits were identified using the docking‐based virtual screening. The compounds inhibiting lactate secretion and cancer cell viability were evaluated by Lactate‐Glo Assay and Cell Viability Screening (**Figure** **3**). One of the compounds, CB‐2 (**Figure** **4**A), has shown a significantly inhibitory effect on lactate secretion and striking cytotoxic activity against MDA‐MB‐231 cells, which are 4.2‐ and 55‐fold, respectively, more potent than those of the reported dual MCT1/4 inhibitor diclofenac ^[^ ^11^ ^]^.

### 2.4 CB‐2 Targets the MCT4 Molecule

To provide insights into CB‐2 interacts with the MCT4, we performed docking simulation for binding modes of CB‐2 to the MCT4 Molecule. The predicted binding modes revealed that CB‐2 might bind to a pocket of MCT4 composed by Ser156, Phe243, Tyr332, Gln339, and Glu363. The side chain of Ser156 formed important hydrogen bonds with CB‐2 (Figure 4B). To identify the direct target(s) of CB‐2, we employed a pull‐down approach using biotinylated CB‐2 analogs. A biotin moiety connected to a linker was attached to CB‐2 at three different positions of benzene ring, forming three CB‐2 analogs (Biotin‐CB‐2‐(linker 1–3)) (Figure 4C). A growth inhibition assay was carried out to check whether CB‐2 remains active following biotinylation. Biotin‐CB‐2‐linker 2 and 3 showed a significant cytotoxicity against MDA‐MB‐231 cells with IC50 values of 3.9 × 10^−6^ and 1.2 × 10^−6^ m, respectively, whereas, Biotin‐CB‐2‐linker 1 did not show marked inhibitory effect (Figure 4D). Based on these results, we moved ahead and carried out pull‐down assays using Biotin‐CB‐2‐(linker 1–3) to identify binding targets of CB‐2. The specific enrichment of MCT4 in the Biotin‐CB‐2 fractions were confirmed by western blot analysis (Figure 4E). We next tested whether CB‐2 shows anti‐tumor effects against human cancer cell MDA‐MB‐231‐driven xenograft tumors in nude mice. CB‐2 strikingly reduced the size and weight of MDA‐MB‐231 tumors (Figure 4F,G) without affecting body weight of the mice (data not shown). Of note, there were no detectable signs of systemic toxicity, implying minimal off‐target or nonspecific effects of CB‐2 in vivo. Combination of CB‐2 and metformin exhibits synergistic antitumor effects.

### 2.5 A Triple Combination of Metformin, Trabectedin, and CB‐2 Strongly Inhibits Breast Cancer Cells

**Figure 5:**
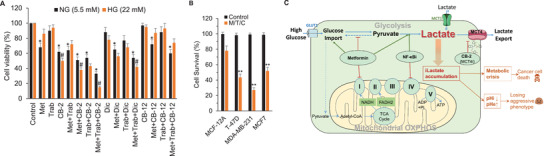
Effects of metformin, trabectedin, and CB‐2 alone or in combination (M/T/C) on the growth of breast cancer cells. A) MDA‐MB‐231 cells were treated with metformin (Met, 1 × 10^−3^ m), trabectedin (Trab, 5 × 10^−6^ m) or CB‐2 (5 × 10^−6^ m) alone and in combination for 96 h under the normal glucose (NG) or high glucose (HG) conditions. DMSO was used as the vehicle for each drug. The growth inhibition was detected by MTT assay. Each value represents the mean ± SD (*n* = 3). Statistical analysis using ANOVA with the Tukey‐Kramer multiple comparison test showed that the number of viable cells was significantly lower in the Met+Trab+CB‐2 group than in the control or any other treatment groups. *, *p* < 0.05 versus Control; #, *p* < 0.05 versus the corresponding NG group. B) Effects of the M/T/C treatment on the growth of various breast cancer cell lines. The MCF‐12A, T‐47D, MDA‐MB‐231, and MCF7 cells were treated with metformin (Met, 1 × 10^−3^ m), trabectedin (Trab, 5 × 10^−9^ m) or CB‐2 (5 × 10^−6^ m) for 96 h. DMSO was used as the vehicle for each drug. The growth inhibition was detected by MTT assay. Each value represents the mean ±SD (*n* = 3). Statistical analysis using ANOVA with the Tukey‐Kramer multiple comparison test showed that the number of viable cells was significantly lower in T‐47D, MDA‐MB‐231, and MCF7 cells. **, *p* < 0.01. C) Model of the proposed metabolic reprogramming strategy (MRS).

In order to predict the therapeutic effect of the MRS, we determined MRS efficacy by using combination of CB‐2 and two clinical drugs (metformin and trabectedin) in MDA‐MB‐231 cells. Trabectedin was used as an NF‐*κ*Bi. It was approved by the US FDA for the treatment of ovarian cancer ^[^ ^12^ ^]^ and recently was identified as a potent NF‐*κ*Bi with IC50 of 20 × 10^−6^ m ^[^ ^13^ ^]^, which may contribute to its anticancer therapeutic effects. Metformin is a prescription drug used primarily in the treatment of type 2 diabetes. The nonspecific MCT4 inhibitor diclofenac (Dic) was included as a positive efficacy control, and CB‐12, without inhibiting MCT4 function, was included as a negative control. Treatment of MDA‐MB‐231 cells with a Met/Trab/CB‐2 combination (M/T/C) had a stronger inhibitory effect on proliferation than the individual drugs alone or for any combination of two drugs (**Figure** **5**A). Importantly, compared to normal glucose condition, MRS elicits more potent cancer cell‐killing effects under HG condition. In addition, MRS also showed stronger inhibitory effects on cell invasion in MDA‐MB‐231 cells under HG condition versus normal glucose (Figure S5, Supporting Information).

Based on our preliminary studies, we hypothesize that cancer cells that are more dependent on glycolysis are likely more sensitive to this MRS combination treatment. Three different types of human cancer cells, MCF‐7, T47D, and MDA‐MB‐231 cells, which exhibit increasing degrees of dependency on glycolysis ^[^ ^14^ ^]^ were employed to compare their susceptibility to the MRS treatment. As shown in Figure 5B, the sensitivity of different breast epithelial cell lines with gradually increasing glycolysis rates (i.e., MCF‐12A, MCF‐7, T47D, and MDA‐MB‐231 cells) to MRS treatment progressively increased. MDA‐MB‐231 is the most sensitive cell line to the MRS treatment and MCF‐12A exhibits the least sensitivity to MRS. Furthermore, compared with MCF‐12A cells, MRS (M/T/C treatment) also has a stronger inhibitory effect on the migration and invasion ability of MCF‐7, MDA‐MB‐231 and T‐47D cells (Figure S6, Supporting Information).

Overall, our preliminary results demonstrate the MRS was feasible by using a NF‐kBi/metformin/MCT4i combination resulting in a successful inhibition of cancer cell proliferation (Figure 5C). We have identified a novel MCT4 inhibitor CB‐2. These studies form the basis for translating our proposed research to a trial for new treatments for TNBC and diabetes‐associated BC.

### 2.6 MRS Treatment Inhibits HG‐Induced Breast Cancer Progression In Vivo

**Figure 6:**
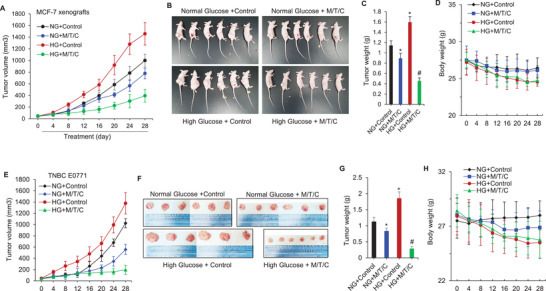
The effectiveness of the MRS treatment in different breast cancer cell lines and animal models of diabetes‐associated BC. A) Dynamic tumor growth of MCF‐7 xenografts isolated from mice treated with vehicle, a single high dose of STZ (200 mg kg^−1^, i.p), CB‐2 (20 mg kg^−1^ day^−1^; i.p) in combination with two clinical drugs, i.e., Trabectedin (0.2 mg kg^−1^, i.p) and Metformin (250 mg kg^−1^ day^−1^; p.o) (M/T/C), or STZ + M/T/C. The M/T/C was administered once every two days for 4 weeks. *n* = 6 mice per group; *n* = 2 independent experiments. B) Representative images from mice treated as (A). C) Tumor weights from B. Values represent mean ± SD; *p < 0.05 versus control; #*p* < 0.05 versus NG+M/T/C. D) Mouse body weights from B. E) Dynamic TNBC tumor growth isolated from normal and type 2 diabetic mice treated with vehicle or M/T/C. The animal model of type 2 diabetes was established as described in Materials and Methods. The mice were injected with the triple‐negative mouse mammary carcinoma E0771 cells into the inguinal mammary fat pad. When the tumors attain a size of 50 mm^3^, mice will be randomized into the following drug treatment groups: Control (injected with the solvent), M/T/C under NG conditions and same group setting under diabetic/hyperglycemic conditions (*n* = 6 per group). F) Representative tumor images from mice treated as (E). G) Tumor weights from E. Values represent mean ± SD; *p < 0.05 versus control; #*p* < 0.05 versus NG+M/T/C. H) Mouse body weights from E.

Next, we investigated the in vivo efficiency and stability of the MRS, establishing a therapeutic dose that inhibits the tumor growth, and identifying the possible side effects. Two different diabetes‐associated BC animal models were used, i.e., type 1 and type 2 diabetic BC mice. With regard to the former, MCF7 cells were orthotopically injected into the mammary fat pads in NU/NU female nude mice. E2 pellets were implanted for the MCF‐7 xenograft model. To evaluate the in vivo effects of hyperglycemia on the tumor growth and antitumor efficacy of MRS therapy, we induced diabetes in some nude mice with a single high dose of Streptozotocin (STZ) (200 mg kg^−1^, i.p). Mice with a glucose concentration exceeding 16.7 × 10^−3^ m were considered diabetic. Figure S8 in the Supporting Information illustrated the dynamic blood glucose changes of animals during the whole treatment period. As shown in **Figure** **6**A–C, hyperglycemia significantly increased the growth of MCF‐7 xenografts. M/T/C could effectively inhibit tumor growth under normal and hyperglycemia conditions, and the inhibitory effect under hyperglycemia conditions is more obvious than that under normal glucose conditions. The M/T/C treatment did not affect body weight of the mice (Figure 6D) and there were no detectable signs of systemic toxicity, implying minimal off‐target or nonspecific effects of M/T/C in vivo. The second animal model is Type 2 diabetic C57BL/6 mice injected with the triple‐negative mouse mammary carcinoma E0771 cells. In this animal model, we also observed the therapeutic effect of M/T/C similar to the previous animal model (Figure 6E–H). It is worth noting that the therapeutic effect of M/T/A on TNBC was obviously better than that of MCF‐7 xenografts, consistent with the aforementioned results in vitro (Figure 5B). To further substantiate the efficacy and mechanism of this MRS strategy, we tested the pH value of tumor tissue slices by using fluorescent BCFL‐AM indicator. As shown in Figure S9 in the Supporting Information, M/T/C treatment could dramatically reduce the intracellular pH value in tumor tissues, and this effect is more significant in the case of hyperglycemia.

Taken together, based on all the experimental results, we conclude that: i) diabetic conditions promote the proliferation and progression of breast cancer xenografts in nude mice. ii) MRS strategy elicit more potent anti‐cancer effects under hyperglycemic versus normoglycemic conditions. iii) MDA‐MB‐231 xenograft tumors with higher MCT4 expression and glycolytic rate will be more sensitive to this MRS combination treatment than MCF‐7 xenografts.

## 3 Discussion and Conclusion

In the present study, we have identified CB‐2 as a novel small molecule MCT4i. Furthermore, we demonstrate that the metabolic reprogramming strategy (MRS), which consists of blocking lactate export via MCT4i in combination with promoting lactate production via forcing glycolysis with metformin and NF‐*κ*Bi, shows an effective anti‐breast cancer effect in preclinical studies.

Cancer cells import massive amounts of glucose which are funneled through the overactive glycolysis metabolic pathway. Increased glycolytic flux in cancer cells leads to production of high amounts of lactate ^[^ ^15^ ^]^. The increased intracellular lactate and H^+^ challenge the mechanisms to maintain intracellular pH (pHi) homeostasis. Intracellular acidification has been shown to be cytotoxic via induction of apoptosis ^[^ ^16^ ^]^. To overcome low pHi due to increased rates of glycolysis, cancer cells remove lactate and H^+^ by monocarboxylate transporters (MCTs) and Na‐driven proton extrusion, respectively ^[^ ^17^ ^]^. Four isoforms of MCTs (MCT1–4) have been identified. To manage the secretion of excessively produced lactate and to prevent intracellular acidosis, cancer cells increase expression of their MCTs, particularly MCT4 ^[^ ^18^ ^]^. Higher concentrations of lactate are detected both locally in the tumor's extracellular matrix and distantly in patient's serum ^[^ ^19^ ^]^. As a consequence, the pH of the extracellular space (pHe) of tumors becomes acidic; forming a reversed pH gradient (pHe < pHi) in comparison to normal physiological conditions (pHe > pHi) ^[^ ^20^ ^]^. The relatively acidic pHe enables cancer progression by promoting proliferation, the evasion of apoptosis, metabolic adaptation, migration and invasion ^[^ ^21^ ^]^. This acidic environment also affects drug uptake by cancer cells, leading to chemotherapy resistance ^[^ ^22^ ^]^. Moreover, in the tumor acidic microenvironment, higher levels of lactate provide signaling for angiogenesis by endothelial cells ^[^ ^23^ ^]^. Increased pHi by itself also has effects on cancer cell function such as augmented proliferation ^[^ ^24^ ^]^, promoting cell survival by limiting apoptosis and selective advantages of growth‐factor independent proliferation. Here, we propose a novel strategy for targeting cancer cells, viz. blocking lactate export by inhibiting MCT4 in combination with promoting lactate production by forcing glycolysis. Disrupting MCT4 function leads to an accumulation of intracellular lactate and decreases pHi ^[^ ^25^ ^]^, which rapidly disables tumor cell growth. Forcing glycolysis by metformin and NF‐*κ*Bi augments this response and the efficacy of MCT4 inhibitors, suggesting an attractive combination therapy for high glycolytic rate/MCT4‐expressing malignancies.

NF‐*κ*B coordinates many of the signals that drive cell activation and proliferation during immunity, inflammation, and oncogenesis ^[^ ^26^ ^]^, but whether it regulates the metabolic reprogramming required for cell division during these processes has only recently been investigated. Four studies demonstrated direct involvement of NF‐*κ*B in regulation of glycolysis and respiration for energy production via the transcriptional regulation of metabolic genes hard‐wired to these processes, with profound implications for oncogenesis ^[^ ^9^, ^27^ ^]^. This NF‐*κ*B‐dependent metabolic pathway involves stimulation of oxidative phosphorylation through upregulation of mitochondrial complex IV subunit SCO2 expression, thus diminishing glycolysis. Moreover, hyperglycemia is accompanied by higher activity of NF‐*κ*B signaling within the cells ^[^ ^28^ ^]^. Accordingly, inhibition of NF‐*κ*B causes cellular reprogramming to aerobic glycolysis ^[^ ^9^ ^]^. The metabolic reorganization that results from NF‐*κ*B inhibition overcomes the requirement for tumor suppressor mutation in oncogenic transformation and impairs metabolic adaptation in cancer in vivo. These studies are consistent with our results demonstrating that NF‐*κ*B inhibition dramatically increases lactate secretion and extracellular acidification in BC cells.

Metformin is an orally administered drug for controlling blood glucose in type 2 diabetes ^[^ ^29^ ^]^, and there is interest in “repurposing” the drug for cancer prevention or treatment. The results of the summarized trial report are mixed, either showing no or weak clinical efficiency ^[^ ^30^ ^]^. Here, we sought to exploit the characteristics of metformin in cellular glucose metabolism to reshape the metabolism of cancer cells. Several metabolic effects have been attributed to this drug. Metformin decreases mitochondrial respiration and citric acid cycle activity, resulting in a decrease in ATP production ^[^ ^31^ ^]^. Thus, cells treated with metformin become energetically inefficient, and display increased aerobic glycolysis and reduced glucose metabolism through the citric acid cycle ^[^ ^32^ ^]^. Consequently, due to the increase of AMP:ATP ratio, AMP‐activated protein kinase (AMPK) becomes activated ^[^ ^33^ ^]^. AMPK activation promotes glycolysis by phosphorylating 6‐phosphofructo‐2‐kinase/fructose‐2,6‐bisphosphatase 3 (PFKFB3) ^[^ ^34^ ^]^. Metformin can also induce glucose consumption and lactate production through an AMPK‐independent mechanism ^[^ ^35^ ^]^. Moreover, metformin's effect on decreasing oxidative phosphorylation may result in induction of glycogenolysis and glycolysis in cells ^[^ ^36^ ^]^. Importantly, cancer cells exposed to metformin display a greater compensatory increase in aerobic glycolysis than nontransformed cells, highlighting their metabolic vulnerability ^[^ ^32^ ^]^. Here, we propose a novel therapeutic strategy for treatment of diabetes‐associated breast cancer by exploiting two actions of metformin, namely controlling blood glucose and forcing glycolysis, in combination with lactate export inhibition. In this light, the ability of metformin to elicit synthetic lethality with NF‐*κ*Bi and MCT4i in cancer cells through rapidly and massively increasing the accumulation of intracellular lactate and then specifically poisoning cancer cells (Figure 5C) might be of potential clinical benefit.

## 4 Experimental Section

### Cell Culture, Treatment, and Standard Assays

Human cell lines, MCF‐12A, MCF‐7, MDA‐MB‐231, T‐47D cells were obtained from ATCC (Rockville, MD). These cells were authenticated by Laragen, Inc. (Culver City, CA), by short tandem repeat (STR) profiling and monitoring cell morphology and biological behavior, and tested to exclude mycoplasma contamination before experiments. The normal glucose‐cultured MCF‐7, T‐47D and MDA‐MB‐231 cells were used for experiments after two months of incessant culture in normal glucose (5.5 × 10^−3^ m) medium. After 5 hours of serum starvation, the cells were treated with normal glucose (NG) (5.5 × 10^−3^ m glucose + 17 × 10^−3^ m mannitol) or high glucose (HG) (22 × 10^−3^ m glucose) for 0–48 h. Some of the cells were also incubated with metformin (Met, 1 × 10^−3^ m), trabectedin (Trab, 5 × 10^−6^ m) or CB‐2 (5 × 10^−6^ m) alone and in combination for 96 h. Standard cell culture and immunoblotting analysis, extracellular acidification rate (ECAR), oxygen consumption rate (OCR), intracellular pH, and cell growth inhibition assay were carried out as described previously ^[^ ^37^ ^]^. For more information, see Supplemental Experimental Procedures.

### Western Blotting

Western blot analysis was carried out by standard methods described before ^[^ ^37^, ^38^ ^]^. Densitometry was performed using Scion Image software (Scion Corp., Frederick, MD). Antibodies were obtained from commercial sources listed below. Anti‐MCT1 was purchased from Cell Signaling Technology; Anti‐MCT3 were obtained from Biorbyt; Anti‐tubulin, anti‐MCT2, and anti‐MCT4 were provided by Abcam.

### Transfection and siRNA Gene Silencing

MCT4 siRNAs from Thermo Fisher Scientific, and negative control siRNA from Ambion (Austin, TX) were purchased. For siRNA transfection, BioT transfection reagent (Bioland Scientific LLC) was used as described previously ^[^ ^37a^ ^]^.

### Lactate Secretion Assay

The levels of lactate and their release into the incubation media were determined by using a commercial kit (Lactate‐Glo™ Assay, #J5021; Promega) according to the manufacturer's protocol ^[^ ^39^ ^]^.

### Homology Modeling of Human MCT4 Structure and Virtual Screening

The 3D model of human MCT4 structure was generated using the I‐TASSER On‐line Server (https://zhanglab.ccmb.med.umich.edu/I‐TASSER/) ^[^ ^40^ ^]^. Virtual screening was performed using grid‐based ligand docking with energetics (GLIDE) software (Schrödinger suite 2009) based on the human MCT4 structure model with default docking parameter settings ^[^ ^41^ ^]^. Hydrogen atoms and charges were added during a brief relaxation performed using the “Protein Preparation” module in Maestro with the “preparation and refinement” option, and a restrained partial minimization was terminated when the root‐mean‐square deviation (RMSD) reached a maximum value of 0.3Å in order to relieve steric clashes. Amino acid residues located within 20Å from the centroid of residues of Phe130, Ser156, Phe243, Tyr332, and Glu363, as well as the region within 20Å from the centroid of residues of Trp20, Gln205, Thr349, and Asp439, were defined as the binding pockets within transmembrane segment and intracellular side for docking simulation, respectively.

We then virtually screened seven commercial compound libraries (ChemBridge, ChemDiv, Enamine, InterBioScreen, Life Chemicals, Maybridge and SPECS), including more than 5 million compounds, targeting the binding pocket. All compounds were desalted, neutralized, and parameterized using the OPLS 2005 force field. Then, tautomers and ionization states expected to occur in the pH range of 5.0–9.0 were generated using the “ionize” module. In the docking process, standard‐precision (SP) and extra‐precision (XP) docking were respectively adopted to generate the minimized pose, and the Glide scoring function (G‐Score) was used to select the final pose with the lowest energy conformation for each compound. The top‐ranked compounds with high scores were further visually inspected and then pursued for experimental tests.

### Verification of Compound‐Target Protein Binding

To identify the direct target(s) of CB‐2, we employed a pull‐down approach using biotinylated CB‐2 analogs. A biotin moiety connected to a linker was attached to CB‐2 at three different positions of benzene ring, forming three CB‐2 analogs (Biotin‐CB‐2‐(linker 1–3)) (Figure 4C). A growth inhibition assay was carried out to check whether CB‐2 remains active following biotinylation. We then carried out pull‐down assays using Biotin‐CB‐2‐(linker 1–3) to identify binding targets of CB‐2. Cell lysates for detecting binding proteins were prepared according to the previously described procedures ^[^ ^42^ ^]^ and incubated with Biotin‐CB‐2. After incubation at 4°C for 1 h, proteins associated with Biotin‐CB‐2 were pulled down with streptavidin‐agarose (Thermo Pierce). The bound proteins were eluted with SDS‐PAGE loading buffer, separated with a 4–20% gradient polyacrylamide gel and the specific enrichment of MCT1–4 in the Biotin‐CB‐2 fractions were determined by immunoblotting.

### Animal Model of Diabetes‐Induced Breast Cancer Progression

Two different diabetes‐associated BC animal models were used to investigate the antitumor efficiency of the MRS strategy. (a) immunocompromised diabetic mouse model. MCF‐7 cells (Cell Biolabs, Inc.) (5×10^6^ cells) were orthotopically injected into the mammary fat pads in NU/NU female nude mice. E2 pellets were implanted for the MCF‐7 xenograft model. Diabetes/hyperglycemia in some nude mice were induced with a single high dose of Streptozotocin (STZ) (200 mg kg^−1^, i.p). Mice with a glucose concentration exceeding 16.7 × 10^−3^ m were considered diabetic. One week after the injection of the cells, the animals were inspected periodically for the development of a tumor in the injection site. (b) Type 2 diabetic C57BL/6 mice injected with the triple‐negative mouse mammary carcinoma E0771 cells. We first fed mice with the control (D12329) or diabetogenic diet (D12331) (Research Diets, NJ, USA). After 14 days, the first round STZ was administered (3 times a week at 40 mg kg^−1^). The corresponding diet lasted for another four weeks ‐midway through this, mice were injected with E0771 cells (5E5) into the inguinal mammary fat pad. The diet regimen continued followed by the second round of STZ (3 times a week at 40 mg kg^−1^) and the corresponding diet. Food intake and body weight were measured once a week. Within a week of diabetogenic diet administration, baseline plasma glucose and insulin are significantly increased and stabilized. Glucose levels were monitored using an AccuMeter. When the tumors attained a size of 50 mm^3^, mice were randomized into the following drug treatment groups: Control (injected with the solvent), CB‐2 (20 mg kg^−1^ day^−1^; i.p) in combination with two clinical drugs, i.e., Trabectedin (0.2 mg kg^−1^, i.p) and Metformin (250 mg kg^−1^ day^−1^; p.o) (M/T/C) under NG conditions and same group setting under diabetic/hyperglycemic conditions. The M/T/C was administered once every two days for 4 weeks. *n* = 6 mice/group; *n* = 2 independent experiments. An electronic caliper was used to determine the length and width of each tumor. The equation: volume = length × width ^2^ was used to calculate tumor volumes. At the end of the experiment, the animals were sacrificed and the tumors harvested. All animal studies were performed in accordance with the guidelines approved by the Institutional Animal Care and Use Committee of Charles Drew University of Medicine and Science.

### Statistical Analysis

Statistical analysis in the present study was performed using SPSS version 18.0 software (SPSS Inc.). Results were expressed as “mean value ± SD.” The significance of mean values between two groups was determined by Student's *t* test. All differences were two‐sided. For the in vivo studies, one‐way or 2‐way ANOVA and post‐hoc multiple comparisons were used to determine possible differences among the groups. A *p* value < 0.05 was considered statistically significant.

## Conflict of Interest

The authors declare no conflict of interest.

## Supporting information

Supporting Information

## Data Availability

The datasets used and/or analyzed during the current study are available from the corresponding author on reasonable request.
