# Supplementary material for: A Novel Metabolic Reprogramming Strategy for the Treatment of Diabetes‐Associated Breast Cancer
Source: Adv Sci (Weinh). 2022 Jan 12;9(6):2102303. doi: 10.1002/advs.202102303 (PMC8867195; doi:10.1002/advs.202102303)
Supplement: Supplementary file 1 — Supporting Information [file ADVS-9-2102303-s001.pdf]

ADVANCED SCIENCE
Open Access

Supporting Information

for *Adv. Sci.*, DOI: 10.1002/advs.202102303

A Novel Metabolic Reprogramming Strategy for the Treatment of Diabetes-Associated Breast Cancer

Qiongyu Hao, Zhimin Huang, Qun Li, Dingxie Liu, Piwen Wang, Kun Wang, Jieqing Li, Wei Cao, Wenhong Deng, Ke Wu, Rui Su, Zhongmin Liu, Jay Vadgama, and Yong Wu**

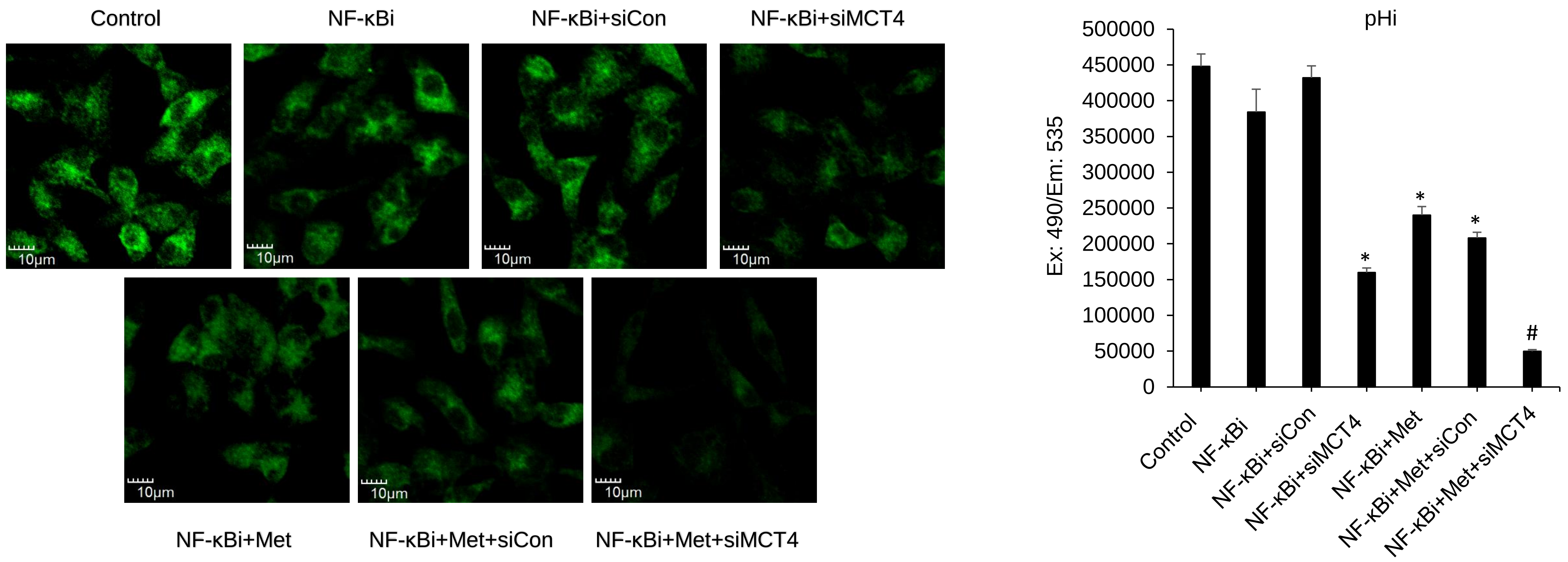

Supplemental Fig. 1 Measurement of pHi level MDA-MB-231 cells. MDA-MB-231 cells were treated with NF-κBi or together with metformin (Met, 0.75 mM) or Control/MCT4 siRNA for 24 h. Intracellular pH was determined with a fluorometric Intracellular pH Assay Kit. Fluorescent BCFL-AM indicator was used to determine pHi of the cells. Each column (right panel) represents the mean ± SD of three independent experiments. *, $P < 0.05$ versus control; #$P < 0.05$ vs. NF-kBi+Met+siCon.

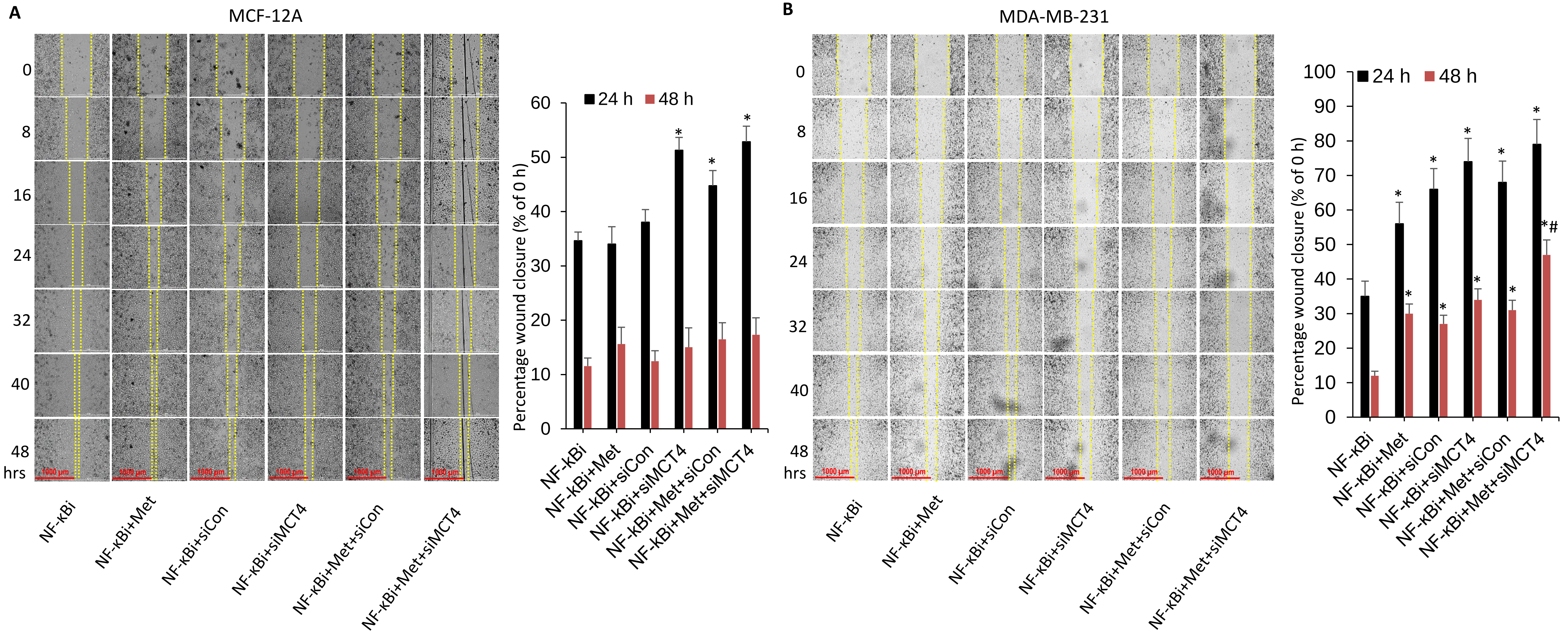

Supplemental Fig. 2 The significant cell migration inhibitory effect of MRS in MDA-MB-231 cells. (A) Timelapse imaging of confluent MCF-12A monolayers cells that were treated as indicated, then wounded by scraping. Cell migration to the wound surface was monitored from 0 to 48 h. The relative wound closure was observed under a microscope and photographed (objective 4×). Scale bars: 1000 μm. Data are presented as the mean ± SD from three technical replicates. *P < 0.05 vs. NF-kBi. (B) Timelapse imaging of confluent MDA-MB-231 monolayers cells that were treated as indicated, then wounded by scraping. Cell migration to the wound surface was monitored from 0 to 48 h. The relative wound closure was observed under a microscope and photographed (objective 4×). *P < 0.05 vs. NF-kBi; #P < 0.05 vs. NF-kBi+Met+siCon.

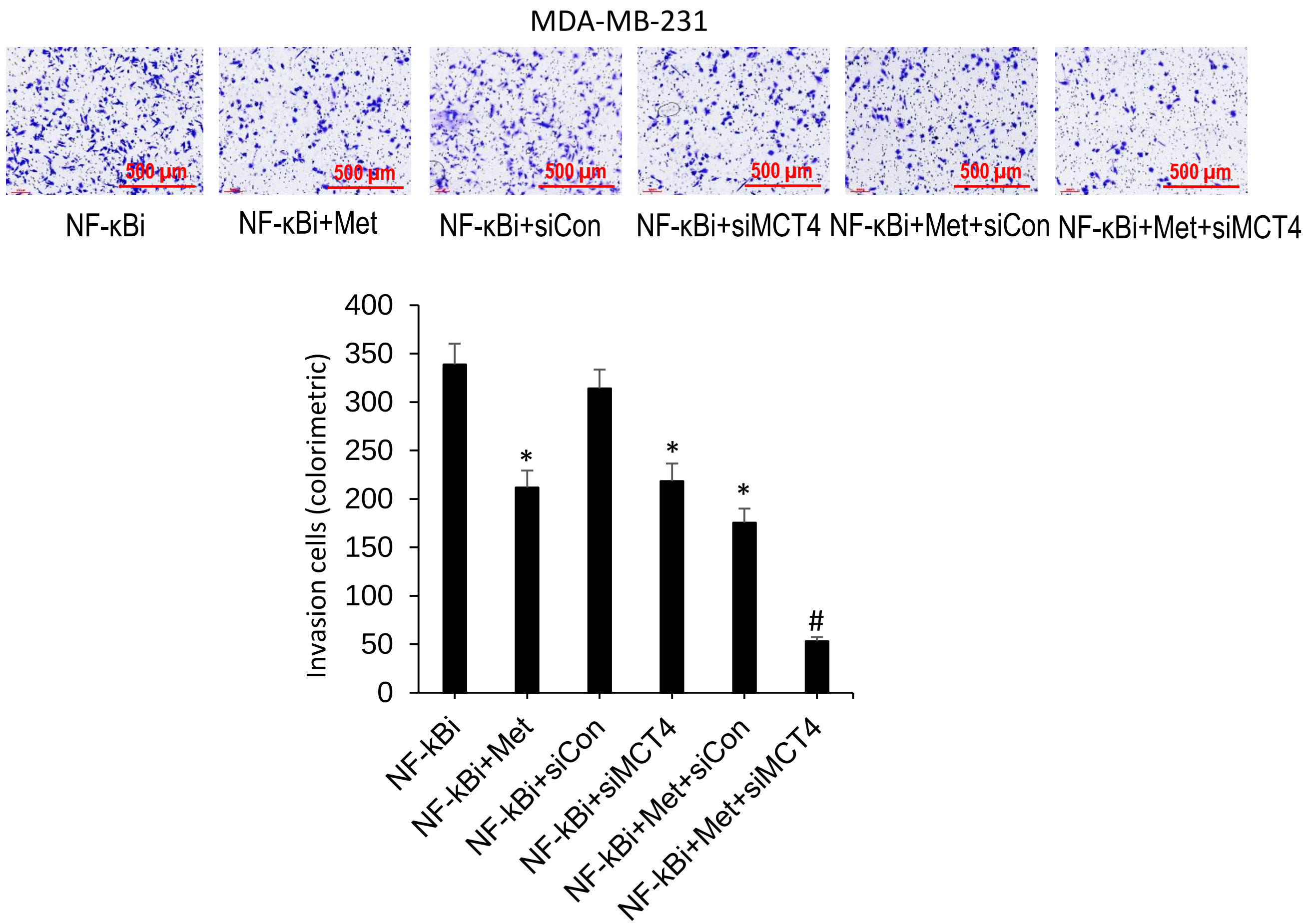

Supplemental Fig. 3 The significant cell invasion inhibitory effect of MRS in MDA-MB-231 cells. Evaluation of the effect of NF-κb, Met, and MCT4 on the invasion of MDA-MB-231 cells by transwell matrigel invasion assays. Each column (lower panel) represents the mean ± SD of three independent experiments. Scale bars: 500 μm. *, P < 0.05 versus NF-kBi; #P < 0.05 vs. NF-kBi+Met+siCon.

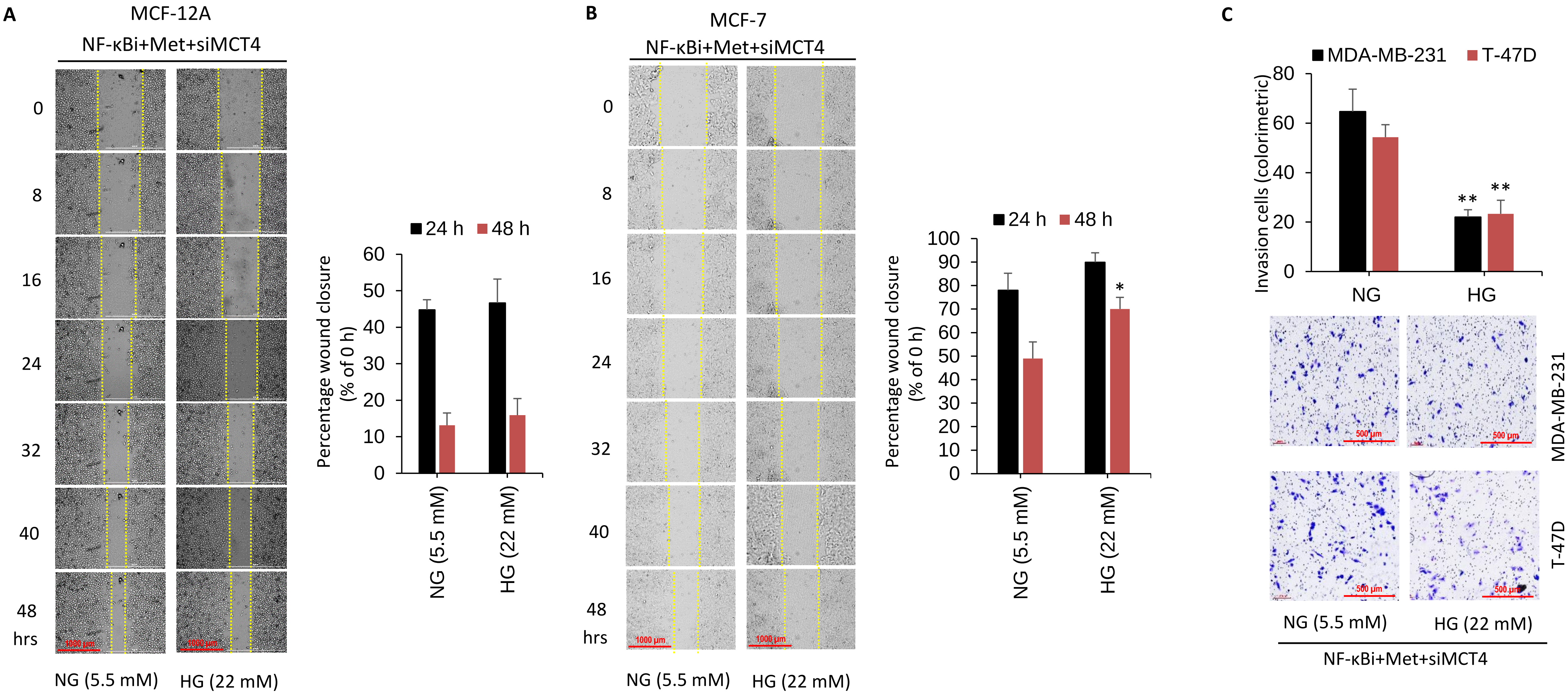

Supplemental Fig. 4 Comparison of effects of MRS (NF-κBi+Met+siMCT4) on cell migration and invasion between normal glucose (NG, 5.5 mM) and HG (22 mM) conditions in different cell lines. (A) Timelapse imaging of confluent MCF-12A monolayers cells that were treated as indicated, then wounded by scraping. Cell migration to the wound surface was monitored from 0 to 48 h. The relative wound closure was observed under a microscope and photographed (objective 4×). Scale bars: 1000 µm. Each column (right panel) represents the mean ± SD of three independent experiments. (B) Timelapse imaging of confluent MCF-7 monolayers cells that were treated as indicated, then wounded by scraping. Cell migration to the wound surface was monitored from 0 to 48 h. The relative wound closure was observed under a microscope and photographed (objective 4×). *, $P < 0.05$ vs. NG. (C) Evaluation of the effect of glucose in combination with NF-κbi, Met, and siMCT4 on the invasion of MDA-MB-231 or T-47D cells. NF-κBi, Met, and siMCT4 were added to cell culture medium containing normal or high glucose in the upper wells of the chambers as indicated. MDA-MB-231or T-47D cells were seeded into the upper wells. The cells invading to the lower surface of the membrane were examined after 36 h. Scale bars: 500 µm. **, $P < 0.01$ vs. NG.

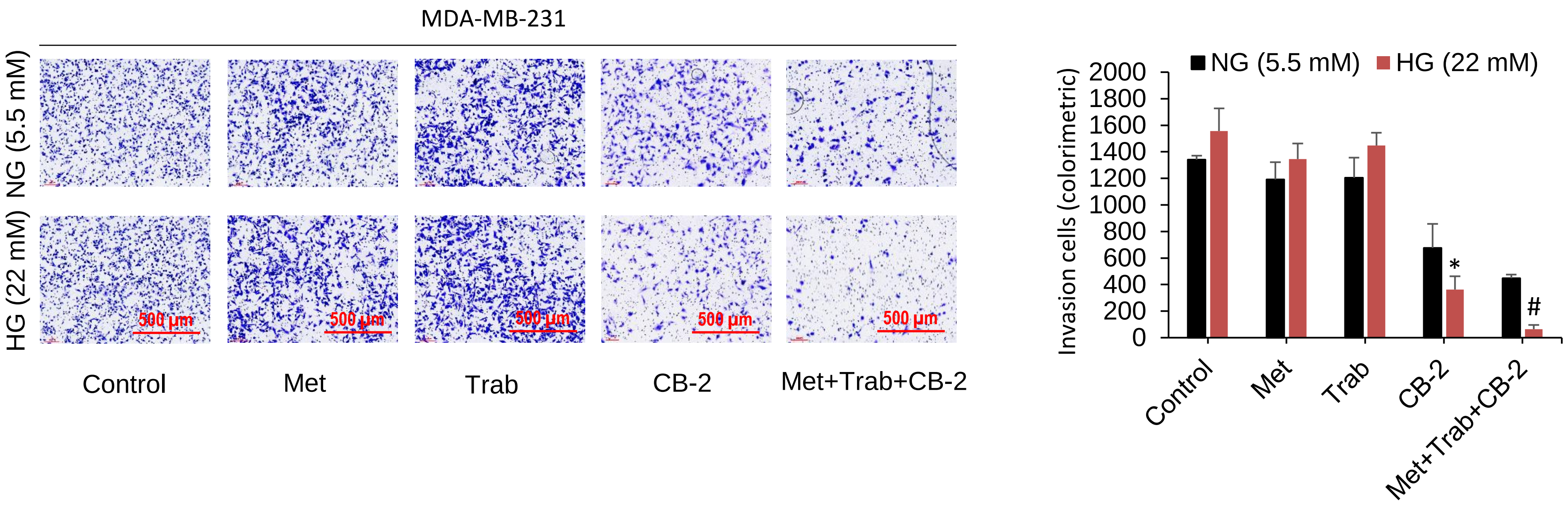

Supplemental Fig. 5 Effects of metformin, trabectedin, and CB-2 alone or in combination (M/T/C) on the cell invasion of MDA-MB-231 cells. In vitro invasion assay performed on MDA-MB-231 cells that treated with metformin (Met), trabectedin (Trab), and CB-2 alone or in combination in the presence of NG or HG. Each column (right panel) represents the mean ± SD of three independent experiments. Scale bars: 500 µm. *, $P < 0.05$ vs. Control; #$P < 0.05$ vs. CB-2.

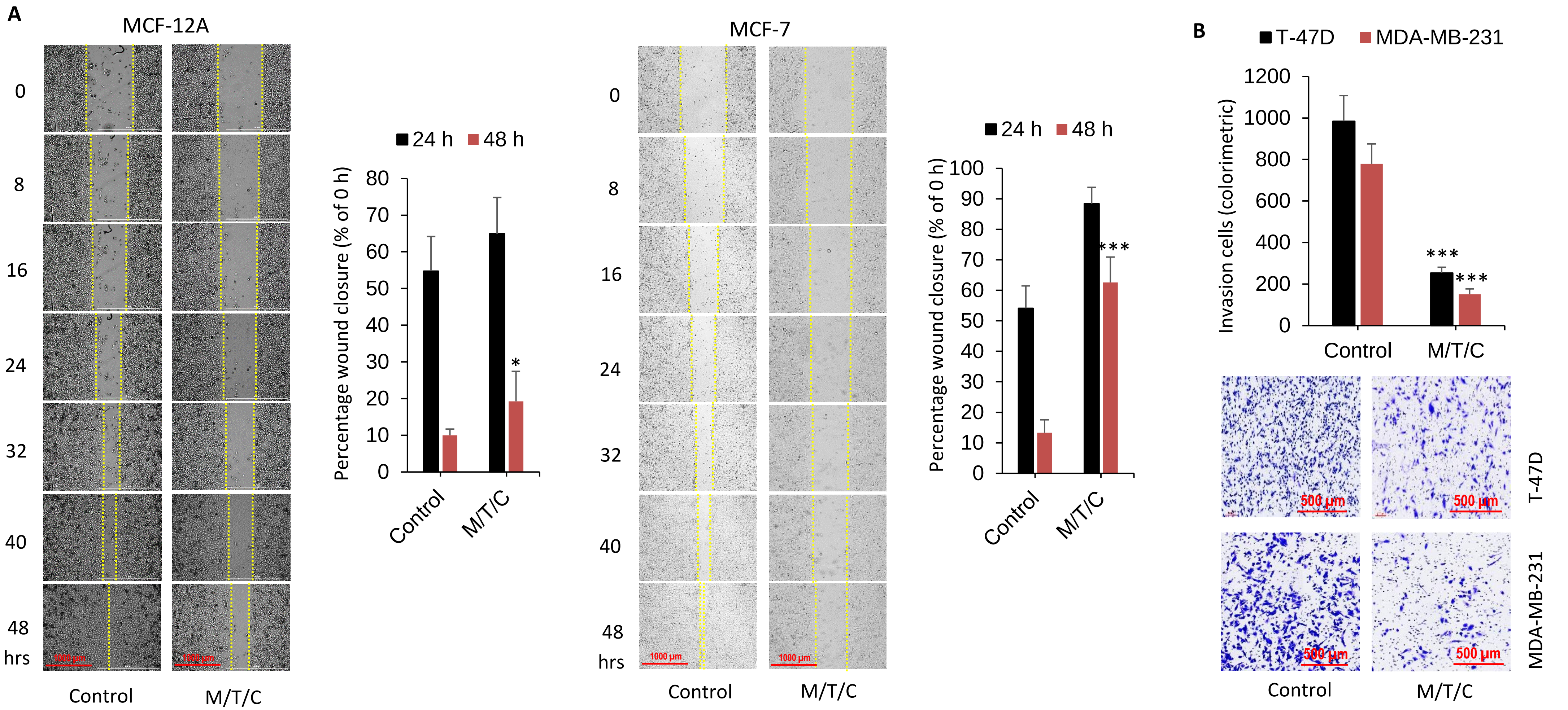

Supplemental Fig. 6 Effects of the M/T/C treatment on the migration and invasion of various breast cancer cell lines. The MCF-12A, T-47D, MDA-MB-231, and MCF7 cells were treated with metformin (Met, 1 mM), trabectedin (Trab, 5 nM) or CB-2 (5 μM) for 24 h. DMSO was used as the vehicle for each drug. The migration inhibition was detected by wound healing assay (A), Scale bars: 1000 μm, and the invasion inhibition was detected by transwell matrigel invasion assay (B), Scale bars: 500 μm. . Each value represents the mean ±SD (n=3). *, p<0.05; ***, p<0.001 vs. Control.

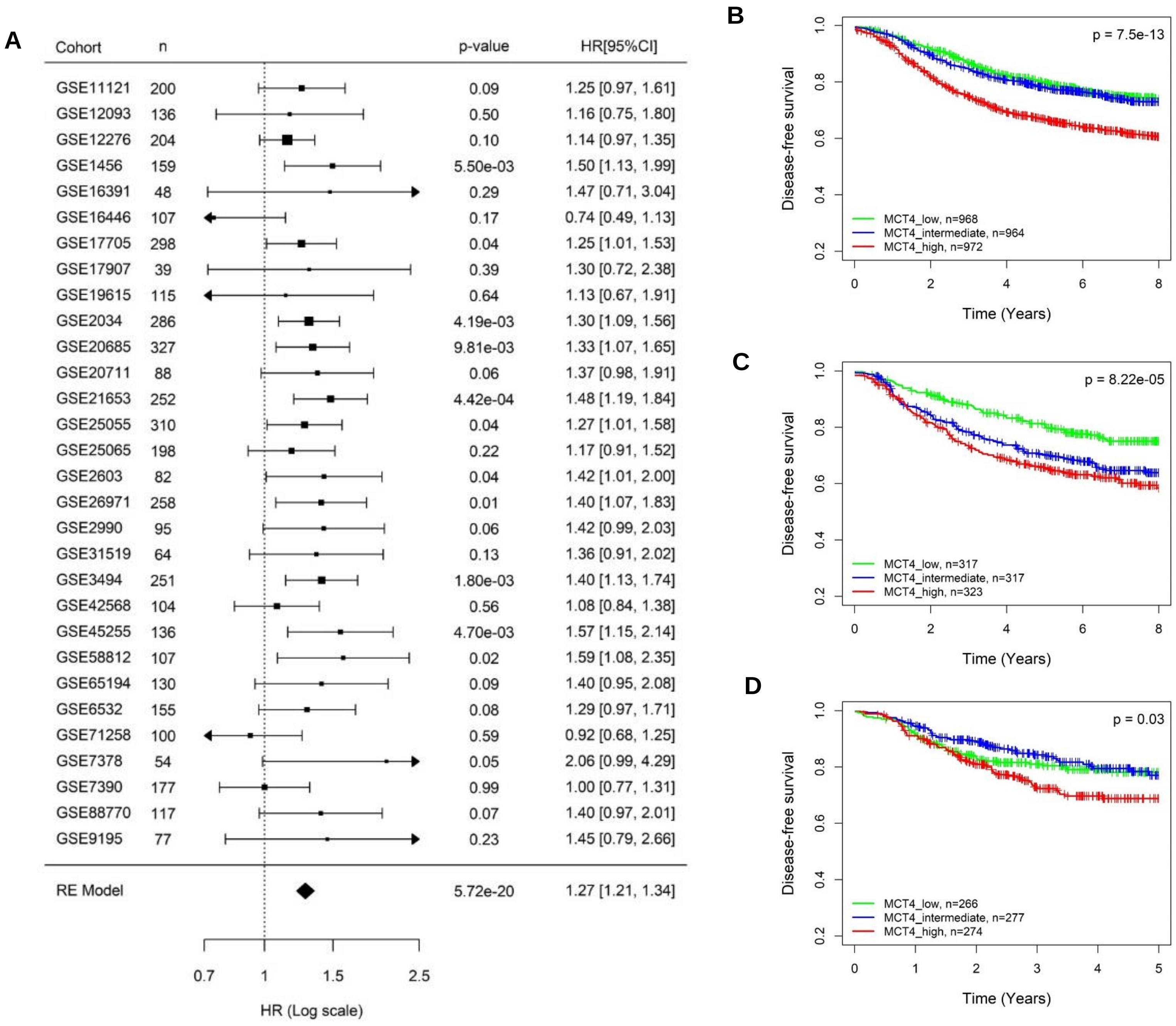

Supplemental Fig. 7 The expression of MCT4 is related to the recurrence risk of breast cancer (BC). (A) Cox regression analysis of the associations of MCT4 expression with recurrence risk of overall BC. MCT4 expression level was used as a continuous variable and a random-effects model was used to calculate the overall prognostic effect in overall BC (30 independent BC datasets). The HRs (presented per one standard deviation increment) are shown in forest plots, in which the squares and horizontal lines represent the HR and 95% CI for the individual datasets, while the diamonds represent the HR and 95% CI for the overall estimate. (B) Kaplan-Meier analysis of the associations of MCT4 expression with recurrence risk of overall BC. Three BC cohorts with survival information were analyzed, including cohort 2 (B), cohort 3 (C) and cohort 4 (D). The patients were stratified into three groups based on tertile splits of MCT4 expression level (high: > 2/3 percentile; low ≤ 1/3 percentile; intermediate: ≤ 2/3 percentile and >1/3 percentile) for each cohort.

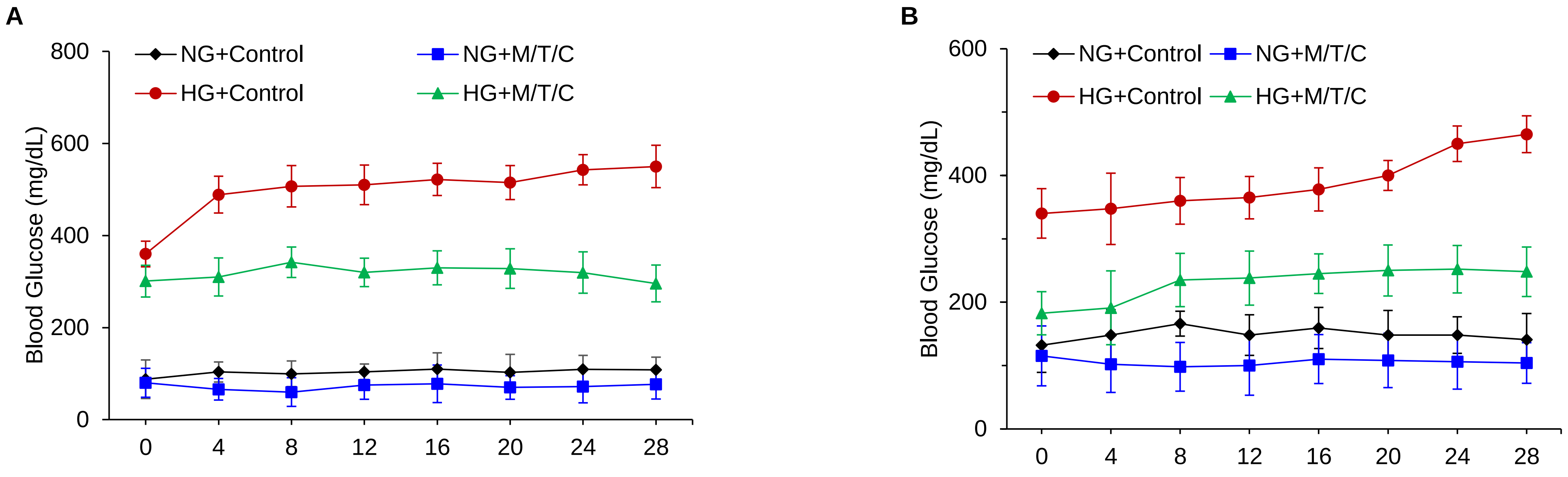

Supplemental Fig. 8 Dynamic blood glucose changes in experimental animals. (A) Dynamic blood glucose levels of MCF-7 xenografts isolated from mice treated with vehicle, a single high dose of STZ (200 mg/kg, i.p), CB-2 (20 mg/kg/day; i.p) in combination with two clinical drugs, i.e. Trabectedin (0.2 mg/kg, i.p) and Metformin (250 mg/kg/day; p.o) (M/T/C), or STZ + M/T/C. The M/T/C was administered once every two days for 4 weeks. n = 6 mice/group; n = 2 independent experiments. (B) Dynamic blood glucose levels from normal and type 2 diabetic mice treated with vehicle or M/T/C. The animal model of type 2 diabetes was established as described in Materials and Methods. The mice were injected with the triple-negative mouse mammary carcinoma E0771 cells into the inguinal mammary fat pad. When the tumors attain a size of 50 mm^3, mice will be randomized into the following drug treatment groups: Control (injected with the solvent), M/T/C under NG conditions and same group setting under diabetic/hyperglycemic conditions (n=6 per group). Glucose levels were monitored using an AccuMeter. Data are presented as mean ± SD.

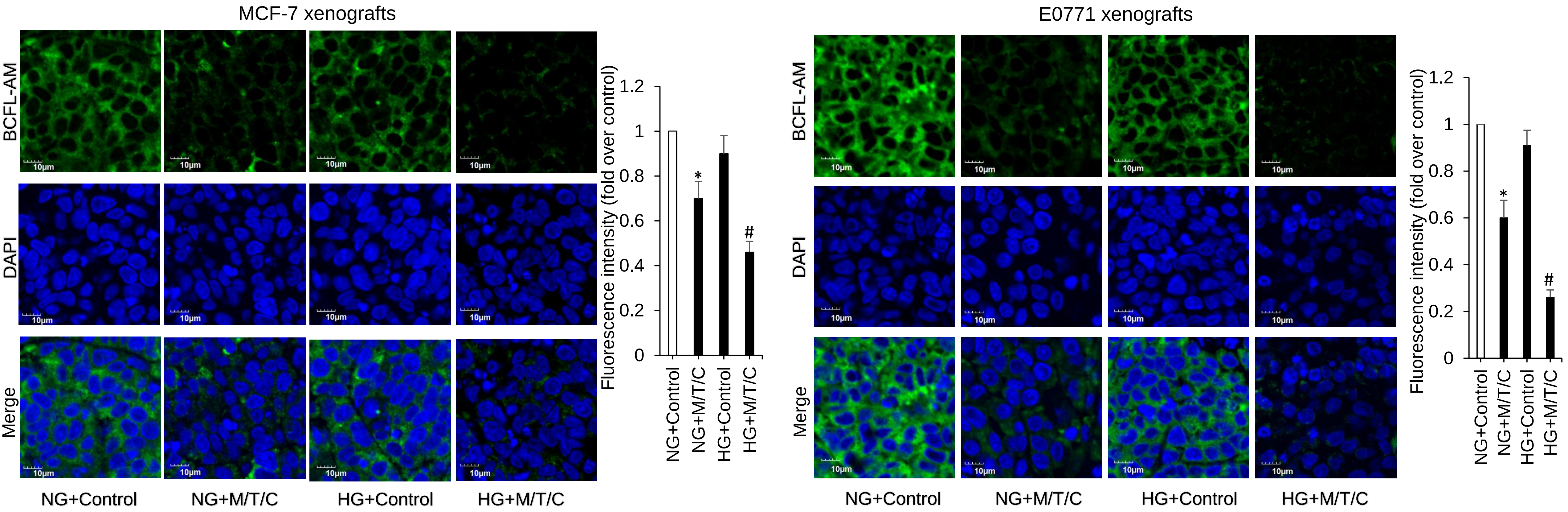

Supplemental Fig. 9 MRS treatment efficiently decreased pHi in the tumor cells from animal models of diabetes-associated BC. Animal models of diabetes-associated BC were established and treated as Fig. 6E. Mice were intraperitoneally injected with BCFL-AM (250 μl) in physiological saline at 5 mg/kg. Frozen cryostat sections (10 μm) were prepared and mounted with anti-fade medium on nonfluorescent glass slides and observed with a fluorescence microscopy. Intensity of fluorescence was quantified with Image-Pro Plus 6.0 software. Values represent mean ± SD; *p < 0.05 versus NG+control; #p < 0.05 versus NG+M/T/C.

Supplemental Materials and Methods

Label-Free Image-Based 2D Scratch Wound Healing Assay

MCF-7 and MCF-12A cells were seeded into 96-well plates and cultured overnight. Once a monolayer of cells had formed, high-quality and consistent wounds across the well in a 96-well plate were automated by an AutoScratch Wound Making Tool (Part Number: AUTOSCRATCH, BioTEK). The displaced cells and cell debris in culture medium were removed and washed out with phosphate buffered saline (PBS), and fresh medium was refilled to allow wound healing. The 96-well plate was placed in a BIOSPA |8 automated incubator (Part Number: BioSpa 8, BioTEK) with a previously set temperature of 37℃ and a CO_2 content of 5%. The wound area was imaged every 8 hours for 48 hours using phase contrast and a 4x objective. Image acquisition is completely automated from sample translation, focusing and exposure control.

Cell invasion assay

Transwell Chamber Assay was used to detect chemotactic invasion assay of MDA-MB-231 or T-47D cells. Cells were seeded in QCM Collagen Cell 24-well Invasion Assay plate (Sigma-Aldrich. Cat#: ECM551) with an 8μm pore size and colorimetric detection was performed. The serum-free DMEM or RPMI 1640 medium was added in the upper inserts and the complete DMEM or RPMI 1640 medium (containing 10% FBS) was added into the bottom chamber as a chemoattractant. After 24-36 hours of culture, the non-invading cells on the membrane were removed by a swab, and invaded cells underneath the membrane were fixed with 4% formaldehyde and stained with crystal violet staining solutions according to the manufacturer's instructions. Five visual fields were randomly selected under a microscope to count the number of invasive cells.

Detection of Intracellular pH (pHi)

Intracellular PH was detected with a fluorometric Intracellular pH Assay Kit (Sigma-Aldrich). Fluorescent BCFL-AM indicator (RatioWorks™ BCFL-AM, AAT Bioquest, USA) was used to determine pHi of the cells. The cells were inoculated in a black plate and incubated for 24 hours. The culture medium was replaced by BCFL-AM reagent, which was prepared in 100 μl HBS solution according to the protocol of the assay kit; subsequently, the cells were incubated in an atmosphere of 37 ℃ and 5% CO2 for 30 minutes (avoiding light). The measurement was carried out at a wavelength of 490 nm (excitation) and 535 nm (emission) in fluorescence analysis (Spectramax M5, BioTek,VT, USA).

For fluorescence imaging reflecting intracellular PH value, MDA-MB-231 cells grown on cover slides were incubated with 2 mM BCFL-AM at 37°C for 30 min. The cover slides were then placed in an open bath imaging chamber (constant temperature 37°C) containing HEPES-MEM without $HCO3^-$. The chamber was mounted on the platform of the Olympus inverted epifluorescence (Olympus fv1200, Japan), and the recording wavelengths are 490 nm (excitation) and 535 nm (emission).

Measurement of fluorescence intensity of intracellular PH value in animal tissue sections

Mice were intraperitoneally injected with BCFL-AM (250 μl) in physiological saline at 5 mg/kg. Twenty minutes later, the dye-loaded tumor tissues were cut into strips with a shaving blade, and the tissues were fixed in an O.C.T compound (Miles, Elkhart, IN) at −20°C. Frozen cryostat sections (10 μm) were prepared and mounted with anti-fade medium on nonfluorescent glass slides (Fisher Scientific) and observed with a fluorescence microscopy (Olympus Fv1200, Japan). Intensity of fluorescence was quantified with Image-Pro Plus 6.0 software.

Microarray datasets of breast cancer

The 30 Affymetrix U133 microarray datasets annotated with patient survival information that were tested in our previous study [1-3] were again used here to examine the associations of MCT4 expression with recurrence risk of BC. The normalization of the raw array data, the elimination of batch effects, and the clinical characteristics of patients in these datasets were described in our previous work [1, 3]. These 30 datasets were further merged into 3 cohorts, named cohort 2, 3 and 4 respectively (cohort 1 merged from other BC datasets that are not annotated with patient survival information and thus are not tested here), for Kaplan-Meier analysis as described previously [1, 3]. Briefly, cohort 2 were merged from 16 of the 30 datasets (including GSE11121, GSE12276, GSE2034, GSE17705, GSE2603, GSE20685, GSE2990, GSE26971, GSE3494, GSE45255, GSE58812, GSE6532, GSE65194, GSE7390, GSE9195, GSE88770) in which metastasis date information is available. Cohort 3 comprises 8 datasets (GSE12093, GSE20711, GSE21653, GSE31519, GSE42568, GSE1456, GSE7378, GSE71258) in which only relapse date information is available. The remaining 6 datasets with RFS or DMFS information (GSE16391, GSE16446, GSE17907, GSE19615, GSE25055, and GSE25065) were merged as the Cohort 4 because the follow-up time of these datasets (about 5 years) was much shorter than that of cohorts 2 and 3. The batch effect in the four merged cohorts was eliminated using the R Combat function, as we showed previously [1]. The disease-free survival time in cohort 2 and cohort 3 was censored at 8 years, while it was censored at 5 years in cohort 4.

Cox proportional hazards regression and Kaplan-Meier survival curves with log-rank test were performed by using R packages including Metafor, Survival, and Survminer.

References

1. Liu D: **AR pathway activity correlates with AR expression in a HER2-dependent manner and serves as a better prognostic factor in breast cancer**. *Cell Oncol (Dordr)* 2020, **43**(2):321-333.

2. Liu D, Wu Y: **Association of an anaplastic lymphoma kinase pathway signature with cell de-differentiation, neoadjuvant chemotherapy response, and recurrence risk in breast cancer**. *Cancer Commun (Lond)* 2020, **40**(9):422-434.
3. Liu D: **Identification of a prognostic LncRNA signature for ER-positive, ER-negative and triple-negative breast cancers**. *Breast Cancer Res Treat* 2020, **183**(1):95-105.
